# A gene missense mutation in diffuse pulmonary lymphangiomatosis with thrombocytopenia: A case report

**DOI:** 10.1097/MD.0000000000021941

**Published:** 2020-09-25

**Authors:** Guixian Zheng, Haijuan Tang, Rui Su, Yi Liang, Zhiyi He, Jianquan Zhang, Jingmin Deng, Jing Bai, Xiaoning Zhong

**Affiliations:** a Department of Respiratory Medicine, First Affiliated Hospital of Guangxi Medical University, Nanning; b Department of Respiratory Medicine, Fourth Affiliated Hospital of Guangxi Medical University, Liuzhou, Guangxi, China.

**Keywords:** lung, lymphangioma, monoclonal antibody D2-40, gene, thrombocytopenia

## Abstract

**Introduction:**

Diffuse pulmonary lymphangiomatosis (DPL) is a rare condition. Most patients with DPL present dyspnea, cough, expectoration, and hemoptysis. There are few reports of DPL accompanied by thrombocytopenia, whose cause remains unknown.

**Patient concerns:**

An 18-year-old male patient presented with recurrent cough, expectoration, and dyspnea for 5 years, and thrombocytopenia was observed during a 2-month follow-up.

**Diagnosis:**

Chest computed tomography showed diffuse patchy shadows in both lungs, and pleural and pericardial effusions. Immunohistochemical lung tissue staining showed lymphatic and vascular endothelial cells positive for D2-40, CD31 and CD34. Routine blood test revealed platelets at 62 × 10^9^ cells/L during follow-up. Bone marrow biopsy was normal. Ultrasound revealed no hepatosplenomegaly. Finally, the patient was diagnosed with DPL accompanied by thrombocytopenia.

**Interventions:**

He was treated by subtotal pericardial resection, thoracocentesis, and anti-infective therapy. Oral prednisone was administered for 2 months.

**Outcomes:**

The symptoms of cough and shortness of breath were improved, but thrombocytopenia persisted. We investigated the cause of thrombocytopenia. Whole-exome sequencing identified a mutation in exon 3 of the *TNFRSF13B* gene in this patient.

**Conclusion:**

DPL may present with thrombocytopenia and DIC. Patients with thrombocytopenia but not DIC and splenomegaly should be screened for gene mutations.

## 1 Introduction

Lymphangiomatosis may occur in single or multiple organ sites. Diffuse pulmonary lymphangiomatosis (DPL) is a rare lung disease characterized by the proliferation of congenital lymphatic vessels, often accompanied by the involvement of lungs, pleura and mediastinal soft tissues. The main chest computed tomography (CT) findings in DPL are thickening of the pulmonary lobule septum, diffuse funicular shadows, reticular opacity, and patchy shadows. The major symptoms of DPL are dyspnea, cough, expectoration and hemoptysis, and seldom thrombocytopenia. To date, 8 cases of DPL have been reported with thrombocytopenia and DIC.^[1–9]^ In addition, only thrombocytopenia was observed in 1 patient^[3]^ (Table 1). Here, we present the case of a young male with DPL involving the mediastinum and pulmonary interstitium, accompanied by thrombocytopenia. He had no splenomegaly and disseminated intravascular coagulation (DIC). We investigated the cause of thrombocytopenia, and whole-exome sequencing was performed on the patient samples. The identified mutation could be an alternative explanation for DPL with thrombocytopenia.

**Table 1:**
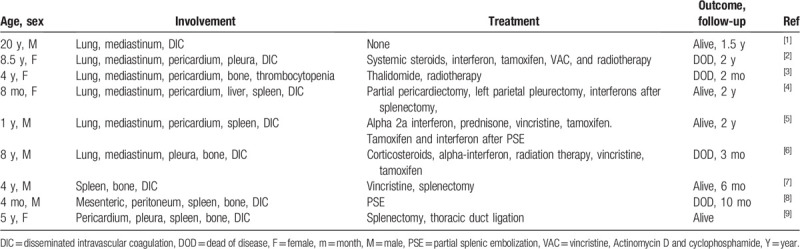
Nine cases of lymphangiomatosis with thrombocytopenia.

## 2 Case presentation

**Figure 1:**
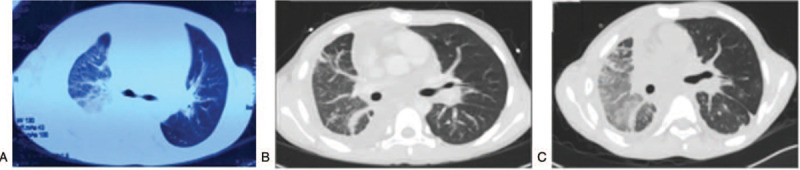
(A) Chest computed tomography (CT) in April 2012 showing a massive right pleural effusion, as well as patchy, dense patches. (B) Chest CT in February 2013 showing the collapse of the right thorax and left lower lung, and thickening of the lobular septum. (C) Chest CT in September 2017 showing the collapse of the right thorax; the texture of both lungs was disordered, and glassy density of the right lung was observed in all layers.

The study was approved by the local Ethics Review Board at First Affiliated Hospital of Guangxi Medical University, China (number 2019KY-E-079), and performed according to the Declaration of Helsinki. On February 19, 2013, a 12-year-old boy of Han nationality was referred to our hospital (Department of Pediatrics, the First Affiliated Hospital of Guangxi Medical University) for shortness of breath accompanied by cough and fever. He had been admitted to Liuzhou People's Hospital (a third-class A hospital) after diagnosis of chronic pericarditis and capillary hemangioma 11 months previously. Then, he presented with cough and shortness of breath; chest CT showed large amounts of pleural effusion in the right side of his chest (Fig. 1A), and echocardiography revealed large amounts of pericardial effusions. His condition was improved after subtotal pericardial resection, right chest clearing, and postoperative anti-infective treatment with flucloxacillin, cefoperazone sulbactam, and meropenem. Pericardial pathology reported chronic pericarditis and capillary hemangioma. Half a month ago, he experienced shortness of breath and cough with yellow-white sputum; body temperature raised to 38.5°C irregularly. After treatment with ceftriaxone, the temperature decreased to the normal level. Shortness of breath steadily progressed and was more serious in the lying position. The patient was the product of an uncomplicated pregnancy and delivery, and had experienced no developmental or growth problems. His medical history showed no abnormalities. No family history of lymphedema, bleeding tendency, or vascular malformations was present. The boy was referred to our hospital for further evaluation.

On physical examination, he had a 12 cm old surgical scar on the front of the chest, and the right thoracic cage bulged slightly, whereas the intercostal space was widened. No cutaneous nevi or lymphangiomas was evident. There were right lung percussion, thick bilateral lung breathing, weak bilateral lower lung breathing, and medium and small vesicle sounds, with no pleural frictional sound. His cardiac sounds were normal and no murmurs were present. He had no abdominal masses, hepatomegaly, or splenomegaly. No peripheral edema was present, and neurological examination was normal.

**Figure 2:**
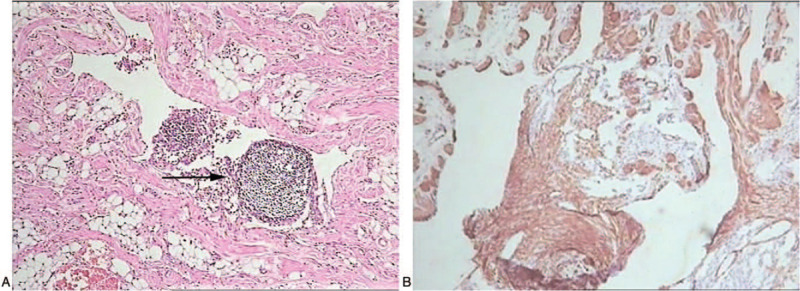
(A) Nine pericardial pathologies showing that the lymphangiomatosis components accounted for 50% to 80% in each slice, with predominant cavernous lymphangiomatosis (original magnification ×10). Lymphocytes were clustered into the bureaucratic cavity (arrowheads) and lined with monolayers and widely spaced endothelial cells. (B) Immunohistochemical detection of D2-40. (DAB, original magnification ×10).

Laboratory examination revealed normal routine blood test, erythrocyte sedimentation rate, tuberculosis antibodies, autoantibody profile, and rheumatoid factor. However, 150 mL of bloody fluid was aspirated by thoracocentesis, and its analysis showed chylous fluid without evidence of infection or malignancy. Pleural fluid cultures for bacteria, fungi, and acid-fast bacilli were negative. Echocardiography revealed minimal pericardial fluid. Chest ultrasound showed bilateral pleural effusion. Reviewed chest CT scan from the local hospital revealed large amounts of pericardial effusion, bilateral pleural effusion, and widening mediastinum. However, plain and enhanced chest CT scans showed no abnormalities in the mediastinum (Fig. 1B). A total of 9 pericardial pathologies were reviewed at Liuzhou People's Hospital (Fig. 2A and B), revealing a vascular tumor that included lymphangioma and hemangioma. Lymphangioma accounted for 50% to 80% of each section, and the morphology mainly reflected cavernous lymphangioma. Immunohistochemical staining showed that proliferating lymphatic endothelial cells expressed D2-40 and CD31. Vascular endothelial cells were positive for CD34. Peripheral lymphatic smooth muscle cells were positive for desmin and SMA. HMB45, Melan-A, and CD117 staining results were negative, ruling out angiomyolipoma and lymphangiomyoma. Histologic differentiation of lymphangiomatosis from hemangiomatosis is not always easy. Based on clinical findings, pathology, and immunohistochemical data, the overall picture favored the diagnosis of lymphangiomatosis.^[3]^ After right side thoracentesis, anti-infective treatment with ceftriaxone and clindamycin, and atomization for cough, no pericardial and pleural effusions were found in ultrasound examinations. His condition was improved and he was discharged on March 13, 2013.

During follow-up, he was admitted to the hospital twice for recurrent episodes of cough, expectoration and shortness of breath in 2015. Routine blood parameters were within the normal limits. These symptoms were improved after anti-infection and thoracentesis. In September 2017, he was treated in Longtan Hospital of Guangxi (third-class hospital) twice for cough, accompanied by chest tightness, and shortness of breath which was more serious in the lying position. No fever, hemoptysis, rash, subcutaneous haemorrhage, and arthralgia were detected. Routine blood test revealed platelets at 62 × 10^9^ cells/L, whereas the total leucocyte number, types of white blood cells, and coagulation data were normal. Pleural effusion was bloody and milky, and bacterial culture was negative. Chest CT showed infiltration in the lungs, bilateral thickening of the pleura, minimal left pleural effusion, and multiple rib abnormalities with lytic areas in the first to fifth thoracic vertebras. After treatment with mezlocillin/sulbactam sodium and metronidazole in combination with left side thoracentesis, cough and shortness of breath remained. In November 2017, the patient presented again to the Department of Respiratory Medicine of the First Affiliated Hospital of Guangxi Medical University. Platelets were 61.40 × 10^9^ cells/L, and coagulation data were within the normal limits. He also tested negative for disseminated intravascular coagulation (DIC). The bone marrow was normal. Abdominal ultrasound revealed no hepatosplenomegaly. Plain and enhanced chest CT scans revealed lung infiltration and bilateral thickening of the pleura, as well as middle and lower lobe bronchial changes in right lung (Fig. 1C). However, DPL is rare, and we lacked related treatment experience. He was treated with systemic oral corticosteroids (30 mg/day for 2 months). The symptoms of cough and shortness of breath were improved, but thrombocytopenia persisted. He stopped taking the medication after 2 months because he worried about possible side effects after prolonged use. Because the patient was asymptomatic and thrombocytopenia had not worsened as assessed by blood tests, we observed him without specific treatment. He has been followed up for >1.5 years, and has not developed dyspnoea on exertion, lymphedema, or bleeding. Furthermore, chest roentgenographic features have remained stable. Persistent thrombocytopenia in this patient requires careful clinical follow-up.

**Figure 3:**
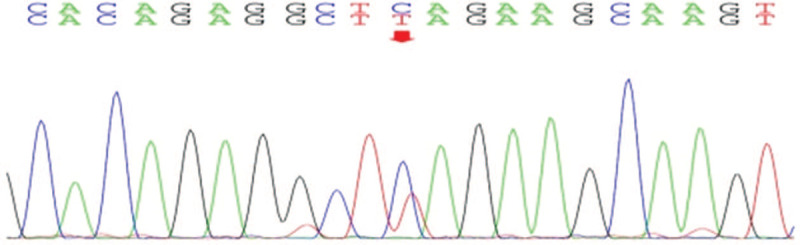
Sanger sequencing of peripheral blood revealed the c.431C > T (p.Ser144Leu) mutation. Arrow indicates the heterozygous C > T mutation at exon 3 of the *TNFRSF13B* gene.

We next investigated the cause(s) of lymphangiomatosis or thrombocytopenia. He is a unique son with no siblings. Whole-exome sequencing by second Generation Exome Sequencing (Beijing Kangxu Medical Laboratory) was performed on samples from his parents. The Review Board of the First Affiliated Hospital of Guangxi University has approved this research. A p.Ser144Leu mutation in TNFRSF13B was found by testing DNA from nucleated cells of the patient's blood, suggesting the possibility of thrombocytopenia (Fig. 3). This mutation was not found in his family members. Lymphatic malformation-related genes previously reported were not found in the current patient.

## 3 Discussion

Lymphangiomatosis is an abnormal proliferation of lymphatic endothelial cells. In 2014, the International Society for the Study of Vascular Anomalies (ISSVA) omitted the terms “lymphangioma” and “lymphangiomatosis” in favor of “generalized lymphatic anomaly”; the literature reflects a change in terminology. These signs commonly present in the head and neck in children. In a 25-year retrospective review of 186 children with lymphangiomas, only 19 patients (10%) had internal or visceral locations, such as the abdomen or thorax.^[6]^ It most frequently involves the liver, the spleen, the mediastinum, lungs, or soft tissues. To date, 49 cases of DPL have been reported. In a review of the literature, we observed that the clinical manifestation of DPL has no specificity. It mainly presents with dyspnea (34/49 [69%]), cough (24/49 [49%]), expectoration (12/49 [24%]), and hemoptysis (10/49 [20%]). Chest imaging in DPL cases also lacks specificity. Many patients (33/40 [83%]) show thickening of the interlobular septum and scattered streak, mesh, and patchy shadows on chest CT images. About 30%∼60% patients present with pleural effusions, occupied mediastinal space, pleural thickening, or pericardial effusions. In the current patient, onset age was 12 years, and he has had DPL for 6 years. The main symptoms were cough, expectoration, and dyspnea. Chest CT mainly showed diffuse patchy shadows in both lungs, and pleural and pericardial effusions.

An important finding in the current patient was the presence of thrombocytopenia without associated splenic lymphangiomatosis. Literature review revealed that thrombocytopenia and DIC are found in a minority of DPL cases (9/49 [18%]). There are currently 9 cases of lymphangiomatosis accompanied by thrombocytopenia. Among them, 8 cases were DIC combined with thrombocytopenia, and 5 had spleen invasion. One case with associated splenic lymphangiomatosis only had thrombocytopenia but not DIC. DIC associated with or without splenic involvement complicates the clinical course. Coagulation abnormalities associated with lymphangiomatosis have been described in reports of thrombocytopenia and DIC. However, there are currently no studies evaluating the cause of thrombocytopenia. DPL with DIC is considered a coagulation abnormality associated with venous malformations; this so-called localized intravascular coagulation could develop into systemic DIC.^[7,10]^ However, this hypothesis lacks a pathological basis, and the actual etiology needs to be further studied. Two cases of DPL involving the spleen with thrombocytopenia and DIC showed improvement after partial splenic embolization and splenectomy; therefore, such cases may be related to lymphoma involving the spleen.^[11,12]^ For the patient in this report, the lesions involved the lungs, the pericardium, and the pleura, but the spleen was not involved; in addition, platelet counts were reduced. A mutation in the *TNFRSF13B* gene (c.431C > T, p.Ser144Leu) was found in the current patient by exome sequencing, and a gain-of-function mutation in TNFRSF13B was reported as a candidate for the predisposition to familial or sporadic immune thrombocytopenia.^[13]^ Therefore, the disease etiology in these patients may be related to gene mutations.

From the literature review, it can be concluded that patients with lymphangioma presenting DIC and thrombocytopenia have high mortality. Among the 9 cases, 4 patients died, and 5 survived. Spleen involvement did not necessarily confer a bad prognosis, as 4 of the 5 patients with splenomegaly survived. Among the 5 surviving patients, 4 underwent surgery, including splenectomy^[4,7,9]^ and splenic artery embolization,^[5]^ and 1 received no specific treatment.^[1]^ Bleeding in the 5 patients after surgery was significantly improved. One patient died after splenic artery embolization for intractable chylothorax and chylous ascites occurred, without DIC recurrence.^[8]^ Systemic steroids, interferons, and radiation therapy exerted only palliative effects. Chemotherapy and tamoxifen, which are potentially effective treatment options, did not change the disease course. Medications (hormones and interferons) and chemotherapy or radiotherapy may play a role in mass reduction, but DIC and thrombocytopenia persisted.^[6]^ Two of the 4 dead patients treated by medication alone had uncontrollable bleeding, and died from respiratory failure and DIC.^[2,6]^ Like the present patient, 1 case with lymphangiomatosis had only thrombocytopenia without DIC and splenomegaly; she was not treated for thrombocytopenia and died from progressive respiratory failure.^[3]^ The current patient had thrombocytopenia during follow-up, but no DIC or spleen involvement; he had no bleeding symptoms or progressive respiratory failure, and received no therapy for thrombocytopenia. Fortunately, he showed no deterioration during follow-up. Moreover, a case was reported with DIC and thrombocytopenia, but no spleen invasion. The latter had no treatment for clinical symptoms, and was in good condition during follow-up.^[1]^ From the literature review, in case of related clinical symptoms, active treatment should be performed. Use of medication alone cannot control bleeding, but splenectomy or splenic embolization is effective for thrombocytopenia and DIC.

Overall, DPL may present with thrombocytopenia and DIC. A wider appreciation of this multisystem disease is warranted. Thrombocytopenia may be associated with mutated *TNFRSF13B* gene. Splenectomy or splenic embolization is effective for DPL combined with thrombocytopenia and DIC.

## 4 Consent for publication

Informed written consent was obtained from the patient and his parents for publication of this case report.

## Acknowledgments

The authors thank Dr. Zili Lv for assistance with pathological diagnosis. The authors would also like to acknowledge the patient's contribution to this report.

## Author contributions

XXXX.

## References

[1] Takahashi K Takahashi H Maeda K An adult case of lymphangiomatosis of the mediastinum, pulmonary interstitium and retroperitoneum complicated by chronic disseminated intravascular coagulation. Eur Respir J 1995;8:1799–802. 8586141 10.1183/09031936.95.08101799

[2] Tamay Z Saribeyoglu E Ones U Diffuse thoracic lymphangiomatosis with disseminated intravascular coagulation in a child. J Pediatr Hematol Oncol 2005;27:685–7. 16344679 10.1097/01.mph.0000193476.14493.06

[3] Kothari SS Sharma S Bhatt K Recurrent hemorrhagic pericardial effusion in a child due to diffuse lymphangiohemangiomatosis: a case report. J Med Case Rep 2010;4:10.1186/1752-1947-4-62 PMC2831910 20170552

[4] Chen YL Lee CC Yeh ML Generalized lymphangiomatosis presenting as cardiomegaly. J Formos Med Assoc 2007;106: 3 suppl: S10–4. 17493902 10.1016/s0929-6646(09)60359-4

[5] Patton DF Kaye R Dickman P Partial splenic embolization for treatment of disseminated intravascular coagulation in lymphangiomatosis. J Pediatr 1998;132:1057–60. 9627607 10.1016/s0022-3476(98)70412-3

[6] Alvarez OA Kjellin I Zuppan CW Thoracic lymphangiomatosis in a child. J Pediatr Hematol Oncol 2004;26:136–41. 14767208 10.1097/00043426-200402000-00018

[7] Dietz WH Jr Stuart MJ Splenic consumptive coagulopathy in a patient with disseminated lymphangiomatosis. J Pediatr 1977;90:421–3. 839336 10.1016/s0022-3476(77)80706-3

[8] Bader-Meunier B Husseini K Nouyrigat V Partial splenic embolization in lymphangiomatosis. J Pediatr 2001;138:613–4. 10.1067/mpd.2001.113784 11295746

[9] Harada K Ito T Shiota T Chylothorax, splenic lymphangiomatosis, and consumptive coagulopathy after surgical treatment of primary chylopericardium. Am Heart J 1994;127:1633–5. 8197997 10.1016/0002-8703(94)90400-6

[10] Yeager ND Hammond S Mahan J Unique diagnostic features and successful management of a patient with disseminated lymphangiomatosis and chylothorax. J Pediatr Hematol Oncol 2008;30:66–9. 18176185 10.1097/MPH.0b013e318159a55a

[11] Zhao J Wu R Gu Y Pathology analysis of a rare case of diffuse pulmonary lymphangiomatosis. Ann Transl Med 2016;4:114. 27127767 10.21037/atm.2016.03.30 PMC4828737

[12] Laverdiere C David M Dubois J Improvement of disseminated lymphangiomatosis with recombinant interferon therapy. Pediatr Pulmonol 2000;29:321–4. 10738021 10.1002/(sici)1099-0496(200004)29:4<321::aid-ppul13>3.0.co;2-c

[13] Salzer U Chapel HM Webster AD Mutations in TNFRSF13B encoding TACI are associated with common variable immunodeficiency in humans. Nat Genet 2005;37:820–8. 16007087 10.1038/ng1600

